# Female Mice with Selenocysteine tRNA Deletion in Agrp Neurons Maintain Leptin Sensitivity and Resist Weight Gain While on a High-Fat Diet

**DOI:** 10.3390/ijms222011010

**Published:** 2021-10-12

**Authors:** Daniel J. Torres, Matthew W. Pitts, Lucia A. Seale, Ann C. Hashimoto, Katlyn J. An, Ashley N. Hanato, Katherine W. Hui, Stella Maris A. Remigio, Bradley A. Carlson, Dolph L. Hatfield, Marla J. Berry

**Affiliations:** 1 Pacific Biosciences Research Center, School of Ocean and Earth Science and Technology, University of Hawaii at Manoa, Honolulu, HI 96822, USA; lseale@hawaii.edu (L.A.S.); mberry@hawaii.edu (M.J.B.); 2 Department of Cell and Molecular Biology, John A. Burns School of Medicine, University of Hawaii at Manoa, Honolulu, HI 96813, USA; mwpitts@hawaii.edu (M.W.P.); ahashimo@hawaii.edu (A.C.H.); ankatlyn@hawaii.edu (K.J.A.); ahanato@hawaii.edu (A.N.H.); katherinewkhui@gmail.com (K.W.H.); stellamaris.remigio@gmail.com (S.M.A.R.); 3 Molecular Biology of Selenium Section, Mouse Cancer Genetics Program, National Cancer Institute, National Institutes of Health, Bethesda, MD 20892, USA; carlsonb@dc37a.nci.nih.gov (B.A.C.); hatfielddolph@gmail.com (D.L.H.)

**Keywords:** selenium, selenoprotein, *Trsp*, hypothalamus, Agrp neuron, sex differences, diet-induced obesity, leptin resistance

## Abstract

The role of the essential trace element selenium in hypothalamic physiology has begun to come to light over recent years. Selenium is used to synthesize a family of proteins participating in redox reactions called selenoproteins, which contain a selenocysteine residue in place of a cysteine. Past studies have shown that disrupted selenoprotein expression in the hypothalamus can adversely impact energy homeostasis. There is also evidence that selenium supports leptin signaling in the hypothalamus by maintaining proper redox balance. In this study, we generated mice with conditional knockout of the selenocysteine tRNA^[Ser]Sec^ gene (*Trsp*) in an orexigenic cell population called agouti-related peptide (Agrp)-positive neurons. We found that female *Trsp^Agrp^KO* mice gain less weight while on a high-fat diet, which occurs due to changes in adipose tissue activity. Female *Trsp^Agrp^KO* mice also retained hypothalamic sensitivity to leptin administration. Male mice were unaffected, however, highlighting the sexually dimorphic influence of selenium on neurobiology and energy homeostasis. These findings provide novel insight into the role of selenoproteins within a small yet heavily influential population of hypothalamic neurons.

## 1. Introduction

As the global obesity pandemic worsens [1], gaining a better understanding of the molecular mechanisms involved is necessary for developing effective treatments. The hypothalamus of the brain, which regulates energy homeostasis throughout the body, becomes impaired in the obese state and is a potential therapeutic target [2]. In recent years, the role of the essential trace element selenium in supporting hypothalamic function, particularly in response to a high-fat diet (HFD), has begun to come to light [3]. Selenium is used to synthesize selenoproteins, a family of proteins that promote redox balance and support physiological processes such as thyroid hormone metabolism [4] and the inflammatory response [5]. Within the hypothalamus, multiple selenoproteins exhibit high levels of expression that appear to be influenced by changes in nutritional status [6,7]. Mouse models with genetic manipulation of selenoprotein expression targeting the hypothalamus have exhibited significant metabolic repercussions [8,9,10].

Previously, Yagishita et al. [8] demonstrated that broad hypothalamic deletion of the selenocysteine tRNA^[Ser]Sec^ gene (*Trsp*), which is required for selenoprotein biosynthesis, increases the susceptibility to diet-induced obesity (DIO) in mice. Knockout of *Trsp* in rat-insulin-promoter (RIP)-positive cells induced oxidative stress and resistance to insulin and leptin, highlighting the critical nature of hypothalamus-resident selenoproteins in energy homeostasis. Conditional *Trsp* knockout was noted to affect a wide range of neuronal cell types, including anorexigenic pro-opiomelanocortin (Pomc) neurons and astrocytes [8]. Among the cell types not affected were the appetite-stimulating agouti-related peptide (Agrp)-positive neurons, a group of ‘first order’ neurons contained within the arcuate nucleus (Arc) of the hypothalamus that is capable of detecting circulating nutrients and hormones such as leptin. This small neuronal subpopulation releases the inhibitory neurotransmitter γ-Aminobutyric acid (GABA) as well as the neuropeptides Agrp and Neuropeptide y (Npy) to suppress the release of thyrotropin-releasing hormone (TRH) and corticotropin-releasing hormone (CRH), which signal to the pituitary gland to promote energy expenditure from the paraventricular nucleus of the hypothalamus. Due to their unique location at the interface between the brain and the bloodstream and their pronounced influence on energy homeostasis, Agrp neurons have increasingly garnered attention as a potential therapeutic target in treating metabolic disease. To assess the role of selenoproteins in Agrp neurons, we created mice with *Agrp-Cre*-driven knockout of *Trsp*. We report that the loss of *Trsp* from Agrp neurons conferred protection from DIO in a sex-dependent manner.

## 2. Results

### 2.1. In Vivo Metabolic Assessment of Mice with Agrp Neuron-specific Ablation of Trsp

**Figure 1:**
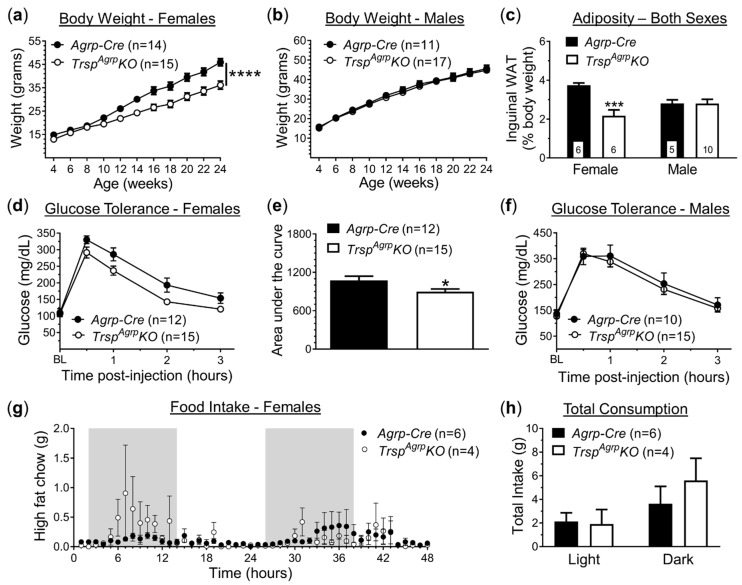
Metabolic profile of *TrspAgrpKO* mice of both sexes raised on a high-fat diet. Female *TrspAgrpKO* mice (**a**) gained less weight while on a high-fat diet compared to *Agrp-Cre* controls; however, there was no effect of *TrspAgrpKO* in the body weight of male mice (**b**) (Sidak’s multiple comparisons test following repeated measures: **** *p* < 0.0001). Inguinal white adipose tissue (WAT) deposits weighed less in female *TrspAgrpKO* mice (**c**) and were unchanged in male *TrspAgrpKO* mice (Tukey’s multiple comparisons test following two-way analysis of variance: *** *p* = 0.0008). Glucose tolerance was elevated in female *TrspAgrpKO* mice (**d**,**e**) compared to controls (unpaired *t*-test comparing area under the curve: * *p* = 0.03) and remained unaffected in males (**f**). Feeding behavior (**g**,**h**) was not significantly changed in *TrspAgrpKO* mice of either sex during 48 h recordings. Samples sizes are displayed in the graphs. All values shown are mean ± standard error of the mean.

To generate mice with Agrp neurons lacking selenoprotein biosynthesis, we crossed *Trsp*-floxed mice with *Agrp-Cre* mice, resulting in mice with a deletion of the *Trsp* gene in Agrp neurons, referred to as *Trsp^Agrp^KO* mice. Control mice consisted of age-matched *Agrp-Cre* mice. Both groups were fed an HFD beginning at 4 weeks of age, after which we monitored body weight and performed metabolic phenotyping. Female *Trsp^Agrp^KO* mice displayed resistance to DIO, gaining roughly 20% less weight than controls by 14 weeks of age (Figure 1a). Weight gained by male *Trsp^Agrp^KO* mice, on the other hand, did not differ from controls (Figure 1b). Female *Trsp^Agrp^KO* mice also displayed reduced adiposity (Figure 1c) and improved glucose tolerance (Figure 1d,e). Consistent with overall body weight trends, male *Trsp^Agrp^KO* mice exhibited similar levels of adiposity (Figure 1c) and glucose sensitivity (Figure 1f) in comparison to control mice.

The primary mechanism through which Agrp neurons control energy homeostasis is the promotion of feeding behavior [11]. Thus, we used specialized metabolic chambers (OxyletPro Physiocage System, Harvard Apparatus) that allow for constant monitoring of single-housed subjects to measure food intake, physical activity, and respiratory metabolism. Although female *Trsp^Agrp^KO* mice were underweight, no significant difference in total food intake was detected compared to controls in either the light or dark phase (Figure 1g,h). Meal analysis revealed a significantly larger inter-meal interval in female *Trsp^Agrp^KO* mice during the light phase (Supplementary Figure S1a), but no changes were observed in any of the other metrics analyzed, which included meal size, meal duration, meal count, and eating rate (data not shown). There were also no detectable changes in physical activity in female *Trsp^Agrp^KO* mice in terms of either total locomotion (Supplementary Figure S1b,c) or rearing events (Supplementary Figure S1d).

**Figure 2:**
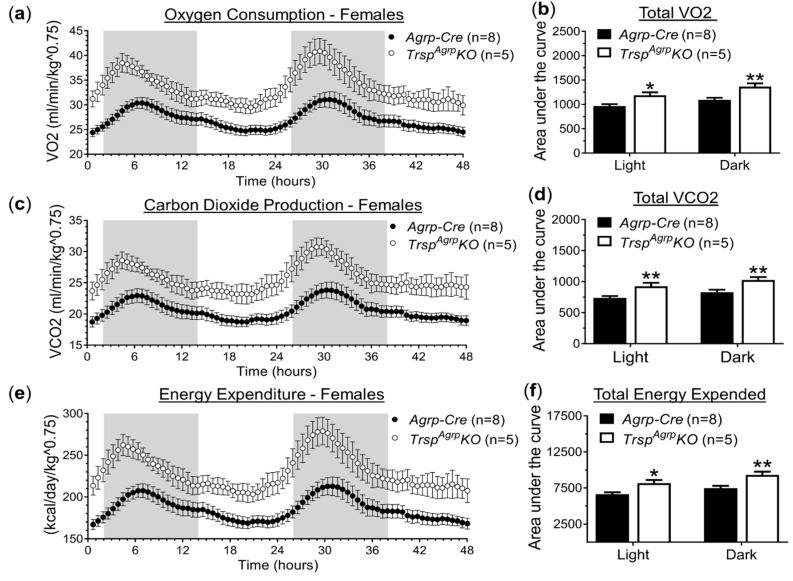
Respiratory metabolism of female *Trsp^Agrp^KO* mice raised on a high-fat diet. Female *Trsp^Agrp^KO* mice displayed elevated oxygen consumption (VO2, volume of oxygen) (**a**,**b**), carbon dioxide production (VCO2, volume of carbon dioxide) (**c**,**d**), and energy expenditure (**e**,**f**) compared to *Agrp-Cre* control mice. Two-way repeated-measures analysis of variance was used to compare the area under the curve during both light and dark cycles. Sidak’s multiple comparisons test: * *p* < 0.05, ** *p* < 0.01. Samples sizes are displayed in the graphs. All values shown are mean ± standard error of the mean.

Since neither feeding behavior nor physical activity seemed to be able to account for resistance to DIO observed in female *Trsp^Agrp^KO* mice, we also probed for changes in respiratory metabolism using the metabolic chamber system. Female *Trsp^Agrp^KO* mice displayed increased rates of oxygen consumption (Figure 2a,b) and carbon dioxide production (Figure 2c,d) compared to control *Agrp-Cre* mice. These differences persisted throughout the duration of both the light and dark cycles. Energy expenditure was estimated using indirect calorimetry and was found to be significantly elevated in female *Trsp^Agrp^KO* mice (Figure 2e,f). Male *Trsp^Agrp^KO* mice did not display any significant changes in respiratory metabolism (Supplementary Figure S1e,f).

### 2.2. Measurement of Hormones in Serum from Trsp^Agrp^KO Mice

Despite having reduced adiposity, female *Trsp^Agrp^KO* mice did not present altered levels of circulating leptin, while insulin levels trended downward (Table 1). Agrp neurons affect the peripheral energy metabolism processes by indirectly suppressing the activity of the pituitary gland. There were no significant differences in serum levels of pituitary hormones between *Trsp^Agrp^KO* and control females. There was a downward trend in follicle-stimulating hormone (FSH) that nearly reached statistical significance, however. Male *Trsp^Agrp^KO* mice had reduced circulating levels of growth hormone (GH), while all other measured hormones were not affected (Table 1).

### 2.3. Histological Analysis of Trsp^Agrp^KO Mouse Brown Adipose Tissue

**Figure 3:**
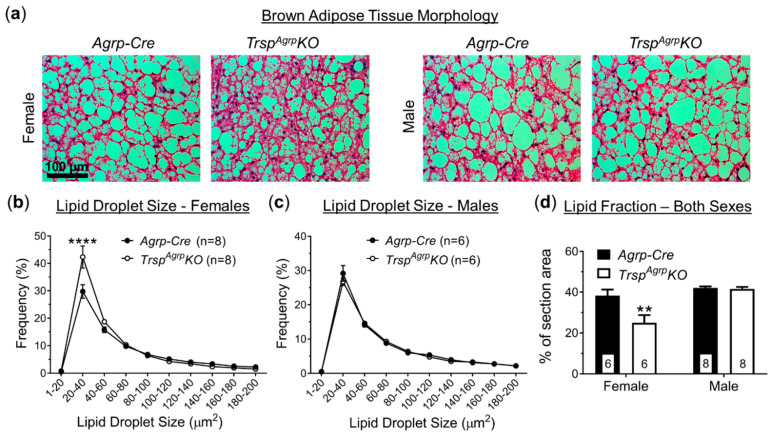
Brown adipose tissue morphology of *Trsp^Agrp^KO* mice of both sexes raised on a high-fat diet. (**a**) Sample images of hematoxylin and eosin-stained brown adipose tissue sections from female and male *Trsp^Agrp^KO* and *Agrp-Cre* control mice. (**b**) The size of lipid droplets measured sections from female *Trsp^Agrp^KO* mice was more frequently present in smaller sizes compared to female *Agrp-Cre* control mice (two-way repeated-measures analysis of variance followed by Sidak’s multiple comparisons: **** *p* < 0.0001). No changes were seen in male mice. (**c**) Average lipid fraction, which is the percentage of the section occupied by lipid droplet, was significantly lower in female *Trsp^Agrp^KO* mice compared to *Agrp-Cre* control mice (**d**) (two-way analysis of variance, followed by Tukey’s multiple comparisons test: ** *p* = 0.007). Samples sizes are displayed in the graphs. All values shown are mean ± standard error of the mean.

In addition to regulating appetite, Agrp neurons are able to influence other metabolic processes, including the thermogenic activity of interscapular brown adipose tissue (BAT) [11]. To evaluate the potential influence of Agrp neuron-specific *Trsp* ablation on BAT thermogenesis, we performed histological analysis of BAT morphology. Lipid droplet size was significantly decreased in BAT sections from female *Trsp^Agrp^KO* mouse BAT, while male *Trsp^Agrp^KO* mice were unaffected (Figure 3a–c). Lipid deposits also occupied a smaller fraction of BAT section surface area on average in female *Trsp^Agrp^KO* mice (Figure 3d). Expression of uncoupling protein-1 (UCP1), a marker of thermogenesis, was not significantly changed in *Trsp^Agrp^KO* mouse BAT sections (Supplementary Figure S2a,b).

### 2.4. Assessment of Leptin Sensitivity in the Hypothalamus of Trsp^Agrp^KO Mice

**Figure 4:**
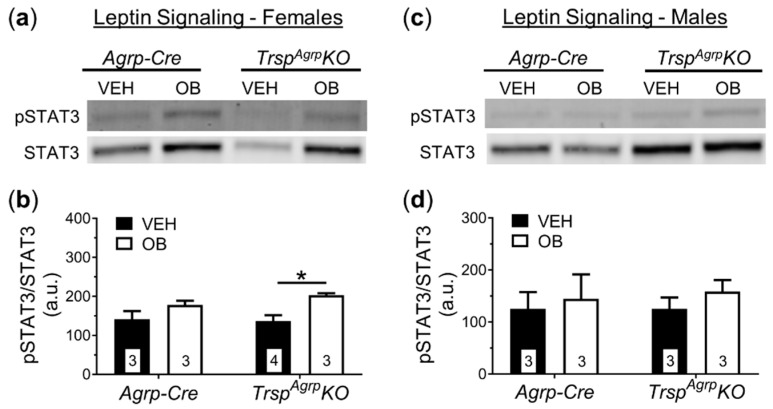
Western blot analysis of broad hypothalamic leptin signaling in *Trsp^Agrp^KO* mice of both sexes raised on a high-fat diet. Leptin (OB) injection did not elicit a significant increase in phosphorylated STAT3 (pSTAT3) protein levels in female *Agrp-Cre* control mice compared to vehicle (VEH)-injected *Agrp-Cre* mice (**a**,**b**). Female *Trsp^Agrp^KO* mice, however, displayed an increase in pSTAT3 levels in response to leptin injection (two-way analysis of variance, followed by Tukey’s multiple comparisons test: * *p* = 0.04). Leptin injection failed to significantly elevate pSTAT3 protein levels in the hypothalamus of either *Agrp-Cre* or *Trsp^Agrp^KO* male mice (**c**,**d**). Samples sizes are displayed in the graphs. All values shown are mean ± standard error of the mean.

To investigate the neural mechanism underlying the DIO resistance phenotype of female *Trsp^Agrp^KO* mice, we performed post-mortem tissue analysis. In response to HFD, the rodent hypothalamus typically develops leptin resistance due to inflammation and oxidative stress [12]. Accumulating evidence suggests that selenoproteins play a major role in regulating hypothalamic leptin signaling [3,13,14]. Therefore, we challenged the mice with intraperitoneal leptin injection prior to sacrifice. Western blot analysis of the whole hypothalamus revealed that female *Trsp^Agrp^KO* mice maintained leptin sensitivity, demonstrated by a significant increase in expression of the leptin signaling protein signal transducer and activator of transcription 3 (STAT3) in response to leptin administration (Figure 4a,b), while control mice developed leptin resistance. As expected, male control and *Trsp^Agrp^KO* mice developed leptin resistance in response to HFD (Figure 4c,d).

**Figure 5:**
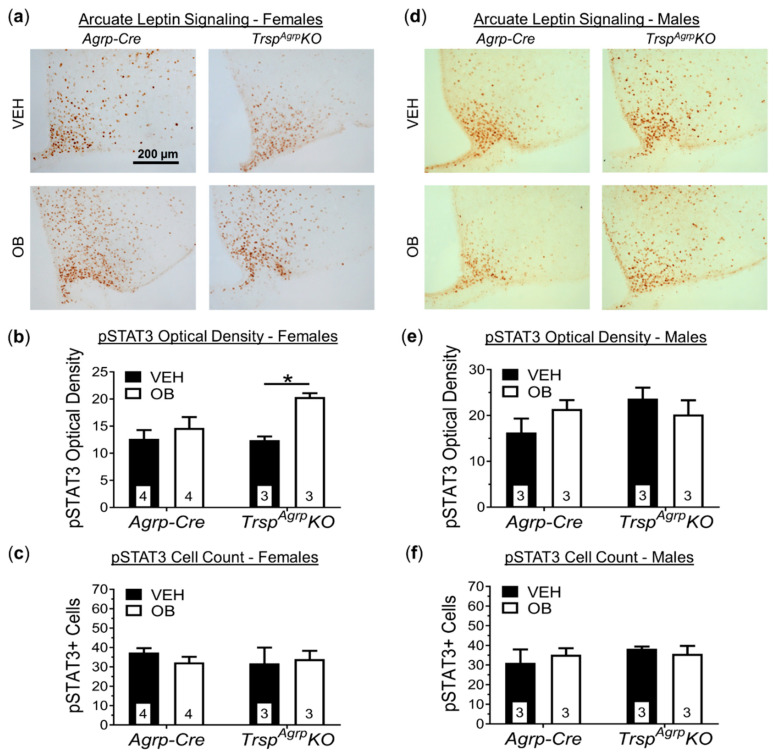
Leptin signaling in brain sections of *Trsp^Agrp^KO* mice of both sexes raised on a high-fat diet. Sample images of hypothalamic sections stained for phosphorylated STAT3 (pSTAT3) following in vivo challenge with either vehicle (VEH, saline) or leptin (OB) (**a**). Images are of the medio-basal hypothalamic area containing the arcuate nucleus and were captured at 20× magnification. Optical density of pSTAT3 immunoreactivity, visualized via 3,3′-diaminobenzidine staining, in the arcuate nucleus of female *Trsp^Agrp^KO* mice was significantly increased by leptin challenge, but not in *Agrp-Cre* control mice (**b**) (two-way analysis of variance, followed by Tukey’s multiple comparisons test: * *p* = 0.03). No change in the number of pSTAT3-positive cells in female *Trsp^Agrp^KO* mice was detected (**c**), however. (**d**) Neither genotype nor leptin administration affected pSTAT3 optical density (**e**) or cell count (**f**) in male mice. Samples sizes are displayed in the graphs. All values shown are mean ± standard error of the mean.

Immunohistochemical measurement of STAT3 expression in hypothalamic sections confirmed that the Arc of the hypothalamus, where Agrp neurons reside, maintains leptin sensitivity in female *Trsp^Agrp^KO* mice (Figure 5a,b). The number of STAT3-positive cells did not increase in response to leptin, however, suggesting a more robust response within a comparable population of leptin-receptor-expressing neurons within the Arc (Figure 5c). Hypothalamic sections from male *Trsp^Agrp^KO* mice showed no significant response to leptin (Figure 5d,e). Importantly, the number of STAT3-positive cells in *Trsp^Agrp^KO* males was comparable to the number present in control mouse sections (Figure 5f).

### 2.5. Analysis of Neuropeptide Expression in the Trsp^Agrp^KO Mouse Hypothalamus

**Figure 6:**
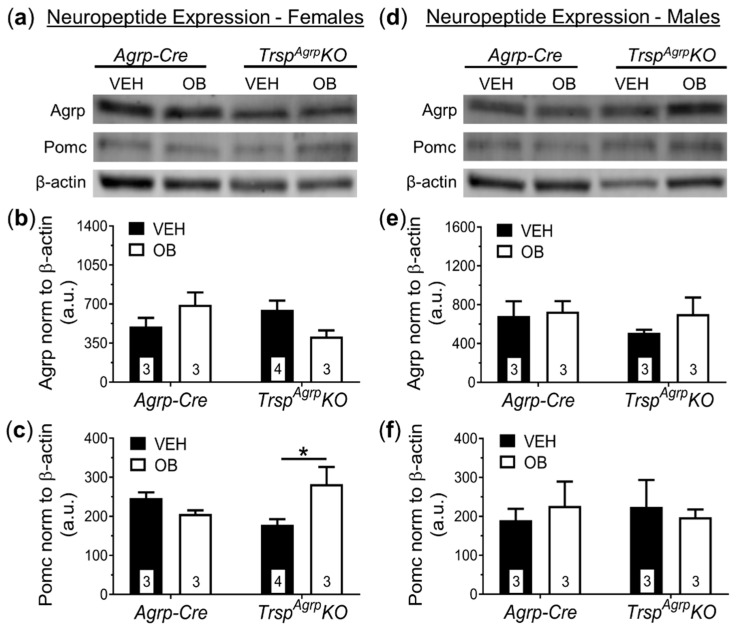
Western blot analysis of broad hypothalamic neuropeptide expression in *Trsp^Agrp^KO* mice of both sexes raised on a high-fat diet. Female *Trsp^Agrp^KO* mice displayed no significant changes in expression of agouti-related peptide (Agrp), but pro-opiomelanocortin (Pomc) was significantly elevated in female *Trsp^Agrp^KO* mice in response to leptin (OB) compared to vehicle (VEH)-injected *Trsp^Agrp^KO* mice (**a**–**c**) (Two-way analysis of variance, followed by Tukey’s multiple comparisons test: * *p* = 0.04). Male *Trsp^Agrp^KO* mice showed no significant changes in Agrp or Pomc protein levels as a result of either genotype or leptin injection (**d**–**f**). Samples sizes are displayed in the graphs. All values shown are mean ± standard error of the mean.

No significant changes in the protein expression of Agrp were observed as a result of *Trsp* deletion in Agrp neurons of female mice (Figure 6a–c). Interestingly, Pomc was upregulated in the hypothalamus of female *Trsp^Agrp^KO* mice in response to leptin (Figure 6c). Male *Trsp^Agrp^KO* mice did not display any significant changes in the levels of hypothalamic neuropeptides (Figure 6d–f).

## 3. Discussion

We report here that the ablation of selenoprotein synthesis via *Trsp* deletion in the Agrp neurons of mice results in a phenotype that is protected against DIO and leptin resistance. This protection was only observed in female mice, however, as male *Trsp^Agrp^KO* mice gained as much weight and adiposity as controls while on an HFD and developed leptin resistance. Female *Trsp^Agrp^KO* mice gained approximately 20% less weight on average than control *Agrp-Cre* mice while on an HFD. Inguinal white adipose tissue (WAT) deposits were about 40% smaller than that found in controls, indicating a leaner body composition, which was accompanied by slightly better glucose sensitivity. Despite the fact that Agrp neurons heavily influence food-seeking behavior, we found that female *Trsp^Agrp^KO* mice consumed similar amounts of food as their control counterparts. The amount of time that elapsed between meals was significantly larger in female *Trsp^Agrp^KO* mice during the light phase, but no other metrics of meal consumption, such as meal size or meal count, were changed. Thus, although there appears to be some modification of feeding behavior, it is unclear whether an increased inter-meal interval in the light phase could account for the 20% decrease in weight gain observed in female *Trsp^Agrp^KO* mice.

It is worth noting that female *Trsp^Agrp^KO* mice were undersized compared to their *Agrp-Cre* counterparts at as early as 4 weeks of age, just prior to HFD exposure (Supplementary Figure S2c). Although these data suggest a developmental growth deficit in female mice due to *Trsp* deletion in Agrp neurons, it is unlikely that it can explain the difference in body weight in adulthood following HFD administration, considering the striking differences in adiposity. Moreover, body lengths measured at 24 weeks of age were not significantly different between groups (Supplementary Figure S2d). A major limitation of the current study is that it did not include an experiment with mice on a control diet. Further investigation that includes a control diet will provide additional insight on this and other aspects of the lean phenotype of female *Trsp^Agrp^KO* mice.

Energy expenditure was found to be significantly elevated in female *Trsp^Agrp^KO* mice. This finding could not be accounted for by a change in physical activity, which remained unchanged, however, suggesting the upregulation of an energy-consuming process within female *Trsp^Agrp^KO* mice. Increased BAT thermogenesis is a potential candidate as lipid depositions were reduced in female *Trsp^Agrp^KO* mice BAT and the influence of Agrp neural activity on thermogenesis is well established [11]. This is consistent with our previous findings in mice with Agrp neurons lacking the selenium recycling enzyme selenocysteine lyase (Scly), *Scly^Agrp^KO mice*, which displayed smaller BAT lipid droplets and increased UCP1 expression in the BAT. We did not observe a change in UCP1 expression in the BAT of *Trsp^Agrp^KO* mice, however, which may suggest a UCP1-independent thermogenic process such as BAT Ca^2+^ cycling thermogenesis or WAT lipolysis [15]. Interestingly, recent studies have depicted the ability of the hypothalamus to increase energy expenditure via sympathetic tone to WAT without affecting BAT UCP1 expression [16].

We did not detect any significant changes in pituitary gland hormones in female *Trsp^Agrp^KO* mice that can explain their resistance to DIO. Circulating levels of FSH, which plays a role in limiting thermogenesis and WAT browning in mice [17], trended downwards in female *Trsp^Agrp^KO* mice, although the change was not quite statistically significant (*p* = 0.05). Interestingly, blocking FSH activates BAT and reduces adiposity in female mice [17]. Since the thyroid-stimulating hormone (TSH) was unchanged, Agrp neurons lacking *Trsp* may be promoting a lean phenotype through a pathway that involves sympathetic innervation rather than endocrine means. In addition to synapsing on the neurosecretory neurons of the paraventricular nucleus, Agrp neurons project to multiple other regions within the hypothalamus. One of these regions, the dorsomedial hypothalamus (DMH), is known to regulate thermogenesis and lipolysis directly via the brain stem [18,19,20]. This DMH-mediated sympathetic pathway is regulated by Npy release from Agrp neurons into the DMH and is thus potentially affected by the genetic deletion of *Trsp*. Therefore, the sympathetic pathway may be involved in the process through which Agrp neurons lacking *Trsp* promote energy expenditure.

Past evidence suggests that selenoproteins play an important role in supporting leptin signaling in the hypothalamus [3]. For example, the endoplasmic reticulum (ER)-resident selenoprotein M (SELENOM) was found to promote intracellular leptin receptor signaling by upregulating the thioredoxin system and protecting against ER stress [13], a known causative factor in diet-induced leptin resistance [21]. Leptin resistance primarily affects the Arc. While the pathology of leptin resistance is not completely known, there is evidence that Agrp neurons develop leptin resistance prior to other cell types, which may serve as an ‘initiating’ event [22]. We therefore expected that *Trsp* ablation would make Agrp neurons more vulnerable to developing leptin resistance. We observed the opposite in female *Trsp^Agrp^KO* mice fed an HFD, however, as the hypothalamus maintained leptin responsivity as a whole and the leptin response in the Arc remained robust. At the surface, it would appear that the loss of the *Trsp* gene prevents Agrp neurons from developing HFD-induced leptin resistance.

The main driver of diet-induced leptin resistance is thought to be hyperleptinemia secondary to excess weight gain [23]. Therefore, since female *Trsp^Agrp^KO* mice remained underweight compared to controls throughout HFD administration, they may not have experienced hyperleptinemia comparable to controls throughout the duration of the HFD regimen. Serum leptin levels of female *Trsp^Agrp^KO* mice were similar to those of female controls, however, despite their lean body composition and heightened leptin sensitivity. One possible explanation for this paradoxical finding is that Agrp neurons in female *Trsp^Agrp^KO* mice develop some level of leptin resistance initially but are replaced by leptin-responsive adult-born neurons [24]. Whereas ablation of Agrp neurons in adult mice causes severe anorexia and rapid starvation [25], genetically induced progressive degeneration of Agrp neurons results in mice that are viable and slightly underweight despite losing ~85% of Agrp neurons by adulthood [26]. Interestingly, according to this report by Xu et al., progressive Agrp neuron ablation caused a decrease in adiposity in female mice, but not males, similar to the results of our study on *Trsp^Agrp^KO* mice. It was eventually uncovered that progressive degeneration of Agrp neurons induces a compensatory mechanism of neurogenesis that includes adult-born Agrp neurons that are responsive to leptin [24]. Past research has shown that global *Trsp* deletion is embryonic lethal [27] and whole-brain neuron-specific knockout of *Trsp* leads to neurodegeneration and seizures [28]. Therefore, it is plausible that the oxidative stress caused by ablating selenoproteins in Agrp neurons may cause a progressive degeneration that induces compensatory neurogenesis similar to that observed by Xu and colleagues [26]. It is important to note, however, that these studies were performed on mice fed a standard diet rather than an HFD, as used in our current study. Interestingly, Pomc protein levels were significantly increased in female *Trsp^Agrp^KO* mice in response to leptin, which may indicate a shift towards greater reliance on Pomc neurons. Whether that is the case and whether those Pomc neurons are new adult-born cells produced in response to Agrp neuron degeneration remains to be investigated.

From the current data, it is not possible to deduce whether the ability of Agrp neurons to avoid leptin resistance drives the lean phenotype of female *Trsp^Agrp^KO* mice or if the adiposity of female *Trsp^Agrp^KO* mice simply does not reach a level necessary to cause leptin resistance in the first place in the time frame used in our study. In the case of the latter scenario, the implication would be that some other factor is driving the lean phenotype of female *Trsp^Agrp^KO* mice. As mentioned above, this could occur in the form of upregulated BAT thermogenesis. Either scenario implies that *Trsp*-lacking Agrp neurons are generally less active than Agrp neurons in control mice as Agrp neurons exert an inhibitory influence on both processes. Indeed, the firing rate of Agrp neurons decreases in response to exogenous reactive oxygen species (ROS) application [29]. Conversely, the anorexigenic Pomc neurons can be activated by ROS [30], suggesting that an oxidative Arc environment promotes a shift towards a negative energy balance. The occurrence of an oxidative suppression of Agrp neuron activity resulting from *Trsp* deletion would reconcile our results with previous reports.

At first glance, our findings may appear to conflict with previous studies that have reported obesogenic phenotypes resulting from models of hypothalamic selenoprotein disruption. For example, broad hypothalamic silencing of selenoprotein synthesis via RIP-Cre-mediated *Trsp* deletion was shown by Yagishita et al. [8] to induce oxidative stress in the hypothalamus and result in an increased susceptibility to DIO accompanied by leptin resistance, insulin resistance, and other metabolic disturbances. It is important to note, however, that the RIP-Cre-driven mouse model used in this study did not appear to affect Agrp neurons specifically, which was the target cell population of the current study. Moreover, the finding that Agrp-specific *Trsp* deletion confers protection against DIO is not surprising, as we have previously reported that *Scly^Agrp^KO* mice are protected against DIO and leptin resistance [10]. The loss of Sclyimpairs the process of synthesizing selenocysteine residues for de facto selenoprotein synthesis, and global *Scly* knockout reduces selenoprotein expression in multiple tissues, including the hypothalamus [31], in addition to producing an obesogenic phenotype [32,33]. We found that Agrp neuron-specific knockout of *Scly* resulted in a lean phenotype similar to, but milder than, what we have observed with the *Trsp^Agrp^KO* mice. While the loss of Scly might result in a reduction in selenoprotein expression, *Trsp* deletion prevents the synthesis of selenoproteins and, presumably, causes even greater vulnerability to oxidative stress. Thus, comparing these two mouse models reveals a potential correlation between the severity of antioxidant incapacitation in Agrp neurons and the extent to which the animal is protected from DIO. It is important to note that there is evidence that alternative amino acids, such as cysteine, can become incorporated rather than selenocysteine [34,35,36,37]. Such a phenomenon might result in non-selenium-containing selenoproteins with diminished functional efficacy. The main difference between the metabolic outcome of these genetic models is that, while both sexes were equally affected by *Scly* deletion in Agrp neurons, only female *Trsp^Agrp^KO* mice were protected from DIO and leptin resistance, whereas any differences between male *Trsp^Agrp^KO* and control mice were barely detectable.

Studies in mice involving selenium and selenoproteins have oftentimes revealed sex differences [33,38,39,40]. To our knowledge, our study is the first to report sex differences in selenoprotein action in the hypothalamus. Although sexual dimorphism within the hypothalamic–pituitary axis has been reported [41], the only potential changes in pituitary-secreted hormones in female *Trsp^Agrp^KO* mice we observed was a decrease in FSH. There are a few studies on hypothalamic physiology involving neurogenesis that might provide clues about the sex differences observed in *Trsp^Agrp^KO* mice. A study by Lee et al. found that an HFD activates neurogenesis in the median eminence (ME) of the hypothalamus of female but not male mice [42]. The researchers found that irradiating the ME, thereby inhibiting neurogenesis, reduced DIO in female mice. This suggests that neurogenic processes mediate DIO in female but not male mice. Therefore, if adult-born Agrp neurons comprise an essential component of this mechanism in female mice, then a lack of selenoprotein action may impair their integration into the local homeostatic circuitry. Interestingly, separate work by Bless and colleagues demonstrated that HFD-induced neurogenesis of leptin-responsive neurons is negatively regulated by estradiol [43] and that an HFD leads to estrogen receptor α (ERα)-positive adult-born neurons [44]. It has also been reported that while Agrp neurons are devoid of ERα expression [45], Pomc neurons express ERα [46]. Therefore, if ERα-positive Pomc neurons are able to exert more homeostatic influence as a result of Agrp neuron degeneration, this may serve to limit neurogenesis-mediated DIO in female mice.

Another possible explanation for the observed sex differences is that the localization of Agrp neurons may contribute to the sex differences in *Trsp^Agrp^KO* mice. One unique quality of Agrp neurons is that a significant portion of them seem to be positioned outside the blood–brain barrier [22]. Moreover, this sub-population seems particularly susceptible to insult and may have a high turnover rate fueled by adult neurogenesis [47]. As mentioned previously, Agrp neurons may develop leptin resistance before other neuronal cell types, and it has been suggested that those lying outside the blood–brain barrier are the first to become desensitized to leptin [22]. If such an event is necessary for the development of leptin resistance, then the ability of female *Trsp^Agrp^KO* mice fed an HFD to maintain leptin sensitivity could be due to these particular Agrp neurons dying or failing to be replaced via neurogenesis before leptin resistance fully develops. It is not clear whether there are any sex differences in Agrp neurons that reside outside the blood–brain barrier. Thus, further investigation of this unique sub-population may provide insight into the origins of the sex differences seen in *Trsp^Agrp^KO* mice. Finally, it is also possible that sexual dimorphism beyond the Arc itself within neural circuitry downstream of Agrp neuron activity may play a role in the sex-specific results obtained from *Trsp^Agrp^KO* mice. Indeed, male and female mice have been found to demonstrate divergence in the sympathetic innervation of adipose tissue [48]. Future studies should unveil the molecular factors and brain circuitry involved.

The results described herein demonstrate the essential nature of selenoproteins in hypothalamic function and energy homeostasis. Remarkably, conditional ablation of selenoprotein synthesis through *Trsp* gene knockout within a small neural population resulted in substantial changes in the ability of mice to gain excess weight while fed an HFD. The sexual dimorphism displayed by *Trsp^Agrp^KO* mice may have implications for the apparent sex differences in lipid metabolism that have been observed in humans, particularly with regard to sympathetic activation of adipose tissue and thermogenesis [49,50]. We believe our findings provide novel insight into the interaction between selenium biology and energy homeostasis. Further investigation of hypothalamic selenoproteins may help identify molecular targets that can be leveraged to develop effective therapeutic treatments for obesity and other metabolic diseases.

## 4. Materials and Methods

### 4.1. Animals

*Trsp^Agrp^KO* mice were generated by cross-breeding Agrp^tm1(Cre)Lowl^/J mice [51] (purchased from The Jackson Laboratory, Bar Harbor, ME, USA) with C57/BL6J mice containing *loxP* sites flanking the gene for selenocysteine tRNA^[Ser]Sec^, designated *Trsp* [52]. Age-matched Agrp^tm1(Cre)Lowl^/J mice, referred to as *Agrp-Cre* mice, were used as controls. Mice were allowed ad libitum food and water access and maintained on a 12 h light/dark cycle. All experiments and procedures were conducted with approval from the University of Hawaii’s Institutional Animal Care and Use Committee (IACUC), protocol: APN 16-2375, last approved 22 September 2021.

### 4.2. Experimental Design

Mice were fed a high-fat diet containing 45% kcal fat and 4.7 kcal/g (Research Diets, New Brunswick, NJ, USA; D12451) beginning at 4 weeks of age. This diet contains ~0.2 ppm of sodium selenite (Mineral Mix S10026). Past studies using this diet resulted in serum total selenium content levels around 0.4 µg/gm measured via inductively coupled plasma-mass spectrometry [32]. Body weight was measured every 2 weeks and measured at 10:00 a.m. Mice were placed in metabolic cages for feeding behavior and respiratory metabolic assessment at 16 weeks of age. At 18 weeks of age, a glucose tolerance test was performed on each mouse, and body weight monitoring continued until mice were sacrificed at age 24 weeks following leptin challenge. Mice were anesthetized either by CO_2_ asphyxiation for fresh tissue harvest or via transcardial perfusion with 4% paraformaldehyde following intraperitoneal injection of tribromoethanol for fixed tissue collection.

### 4.3. Metabolic Chambers

Feeding and drinking behavior, physical activity, and respiratory metabolism were monitored using the PanLab Oxylet*Pro*^TM^ System (Harvard Apparatus, Barcelona, Spain) as previously described [10]. Mice were acclimated to the metabolic chambers for 24 h prior to 48 h of data collection. Oxygen and carbon dioxide concentrations were measured for 7 min periods every 35 min and used to calculate oxygen consumption (VO_2_) and carbon dioxide production (VCO_2_). Data were collected and analyzed using the Panlab METABOLISM software (Vídeňská, Prague, Czech Republic). Energy expenditure (EE) was calculated via indirect calorimetry using the following equation:

EE = (3.815 + (1.232 × RQ)) × VO_2_ × 1.44 (1)

where RQ (Respiratory quotient) is VCO_2_/VO_2_.

### 4.4. Glucose Tolerance Test

Mice were fasted overnight for 16 h. At 10:00 a.m. the next day, blood was accessed via tail vein puncture to measure baseline glycemia. Mice were administered glucose (Sigma; 1 g/kg body weight in sterile phosphate-buffered saline) via intraperitoneal injection. Glycemia was then measured at timepoints of 30 min, 1 h, 2 h, and 3 h post-injection using strips inserted into a glucometer (OneTouch Ultra, LifeScan, Milpitas, CA, USA).

### 4.5. Leptin Challenge and Tissue Collection

On the day of sacrifice, mice were subjected to a leptin challenge. Mice were fasted overnight for 16 h the night before and injected intraperitoneally with leptin (1 mg/kg body weight; R & D Systems, Minneapolis, MN, USA; 498-OB) or sterilized phosphate-buffered saline as a vehicle control. Mice were sacrificed exactly 1 h post-injection. Tissue was either fixed via transcardial perfusion with 4% paraformaldehyde for immunohistochemical analysis or fresh-frozen in liquid nitrogen to be used for Western blots. Prior to perfusion, mice were euthanized with tribromoethanol (1%, 0.1 mL/g body weight), after which blood was collected via cardiac puncture before perfusion with phosphate buffer, followed by 4% paraformaldehyde in phosphate buffer. Brains were collected and stored overnight in 4% paraformaldehyde for overnight fixation, after which they were dehydrated with daily increased sucrose concentrations. Brains were cut into 40 µm coronal sections using a cryostat and stored in floating fashion in a cryoprotective solution (50% 0.1 M phosphate buffer, 25% glycerol, 25% ethylene glycol). BAT was collected and fixed in 4% paraformaldehyde for 1 week, then paraffin-embedded and cut into 5 µm sections. For collection of fresh tissue, mice were euthanized via CO_2_ asphyxiation. Blood was collected via cardiac puncture, and inguinal WAT was removed and weighed on an analytical scale. Brains were placed in 30% sucrose on ice for 1 min, after which the hypothalamus was dissected and immediately frozen in liquid nitrogen.

### 4.6. Gel Electrophoresis and Western Blotting

Frozen tissue was homogenized with a CryoGrinder kit (OPS Diagnostics, Lebanon, NJ, USA; CG 08-01) as previously described [10]. Protein lysate samples containing 40 µg of protein were separated via electrophoresis using a 4–20% gradient polyacrylamide TGX gel (BIO-RAD, Hercules, CA, USA; 5671094) and then transferred onto a 0.45 µm pore size Immobilon-FL polyvinylidene difluoride membrane (Millipore, Burlington, MA, USA; IPFL00010). Membranes were incubated in PBS-based blocking buffer (LI-COR Biosciences, Lincoln, NE, USA; P/N 927) for 1 h, followed by overnight incubation in primary antibody at 4 °C with slow shaking. Blots were washed using PBS containing 0.01% Tween 20 (PBS-T) and incubated with infrared fluorophore-bound secondary antibodies in the dark, washed again with PBS-T, and analyzed using the infrared scanner Odyssey CLx Imaging System (LI-COR Biosciences).

### 4.7. Immunohistochemistry and Histology

For the visualization of target proteins, a 3,3′-diaminobenzidine (DAB) kit was used (DAB Substrate Kit; Vector Labs, Burlingame, CA, USA; H-2200) in conjunction with an avidin-biotin-peroxidase complex (Elite ABC Kit; Vector Labs; PK-6100). Endogenous peroxidases were first sequestered with 1% H_2_O_2_ in methanol, after which sections were blocked in normal goat serum and incubated overnight at 4 °C in primary antibody. Sections were then probed with the appropriate biotinylated secondary antibodies prior to visualization via DAB-mediated reaction. Sections were finally rinsed with phosphate-buffered saline, mounted on glass slides, and dehydrated with an ethanol gradient followed by xylene and cover-slipped.

To visualize BAT morphology, 5 µm sections were stained with hematoxylin and eosin. For measurement of UCP1 immunoreactivity levels, sections were baked in a 60 °C oven for 30 min, deparaffinized using xylene and ethanol, and then incubated in a 1% H_2_O_2_/methanol solution for 30 min. Antigen retrieval was performed using 0.01 M citric acid (pH 6), and sections were blocked with an avidin/biotin blocking kit (Vector Labs; SP-2001), followed by incubation with primary antibody. Sections were then incubated in a biotinylated secondary antibody, followed by DAB-mediated staining and mounting as described above.

### 4.8. Stereology and Data Quantification

Sections were analyzed at bregma −1.46 mm for analysis of the arcuate nucleus. Analysis was performed using the Stereo Investigator Software (MBF Bioscience, Williston, VT, USA) and an upright microscope (Axioskop2; Zeiss, Oberkochen, Germany). For measurement of phospho-STAT3 optical density, a magnification of 20× was used to capture brightfield images which were then imported into ImageJ software for analysis. For quantification, images were first converted into black-and-white, inverted, and then the mean value per pixel was measured within the arcuate nucleus. The Cell Counter ImageJ plugin was used to count the number of phospho-STAT3-positive cells.

For analysis of BAT sections, the simple random sampling function was utilized in Stereo Investigator to capture 20× images. The FIJI (ImageJ v2) plugin Adiposoft was used to count and measure lipid droplet size. To measure UCP1 optical density, images were converted to black-and-white, inverted, and the mean pixel value for the entire image was measured. Assessment of UCP1 optical density was performed as described above for phospho-STAT3.

### 4.9. Measurement of Serum Hormone Levels

Circulating levels of leptin and insulin were assessed by analyzing serum samples using ELISA kits: Mouse Leptin ELISA Kit (Crystal Chem, Elk Grove Village, IL, USA; 90030) and STELLUX Chemiluminescent Rodent Insulin ELISA Kit (Alpco, Salem, NH, USA; 80-INSMR-CH01). Pituitary hormone levels were measured in serum samples using the Mouse Pituitary Magnetic Bead Panel Milliplex Assay (EMD Millipore, Burlington, MA, USA; MPTMAG-49K), performed with the Luminex 200 Instrument System.

### 4.10. Antibodies

The primary antibodies used were: a rabbit anti-phospho-STAT3 (Tyr705) (D3A7) (1:1000; Cell Signaling, Danvers, MA, USA; 9145), a rabbit anti-STAT3 (D1A5) (1:1000; Cell Signaling, Danvers, MA, USA; 8768), a mouse anti-Agrp (1:2000; Alpha Diagnostics, San Antonio, TX, USA; AGRP-11S), a rabbit anti-Pomc (27–52, porcine) (Phoenix Pharmaceuticals, Burlingame, CA, USA; H-029-30), a mouse anti-β-actin (8H10D10) (1:5000; Cell Signaling, Danvers, MA, USA; 3700S), and a rabbit anti-UCP1 (1:500; Abcam, Cambridge, MA, USA; ab10983).

### 4.11. Statistical Analysis

Statistical tests used sample numbers are indicated within each figure legend. Two-way ANOVA, followed by Tukey’s multiple comparisons test, was used to make comparisons using sex and genotype as factors. For data collected over time, including body weight and metabolic cage data, a repeated measures two-way ANOVA was used with Sidak’s multiple comparisons test. An unpaired *t*-test was used to compare glucose tolerance test results. The graphical representations of data reflect the exact statistical comparisons made. For data affected by leptin challenge, comparisons were made within each sex and used genotype and leptin treatment as factors. Data were analyzed, and graphs were generated using GraphPad Prism version 7 software. All data are presented as mean ± standard error of the mean. Significance was determined by a *p*-value of <0.05. Sample sizes represent biological replicates.

## Figures and Tables

**Table 1:** Serum expression levels of hormones *Trsp^Agrp^KO* and *Agrp-Cre* mice.

Hormone Type and Name		*Agrp-Cre*	*Trsp^Agrp^KO*	*p* Value
Females:				
Metabolic hormones				
Leptin (ng/mL)		32.78 ± 1.58 (*n* = 7)	31.54 ± 2.18 (*n* = 9)	0.67
Insulin (ng/mL)		0.54 ± 0.11 (*n* = 7)	0.32 ± 0.04 (*n* = 8)	*0.06*
Pituitary hormones				
ACTH (pg/mL)		20.06 ± 4.69 (*n* = 7)	21.75 ± 4.11 (*n* = 8)	0.79
FSH (ng/mL)		1.41 ± 0.54 (*n* = 6)	0.39 ± 0.11 (*n* = 8)	*0.05*
GH (ng/mL)		1.09 ± 0.43 (*n* = 6)	1.97 ± 0.74 (*n* = 9)	0.39
LH (pg/mL)		125.65 ± 43.95 (*n* = 7)	248.90 ± 76.29 (*n* = 8)	0.20
Prolactin (ng/mL)		9.72 ± 2.10 (*n* = 7)	8.36 ± 1.49 (*n* = 8)	0.60
TSH (pg/mL)		49.40 ± 5.27 (*n* = 6)	61.57 ± 25.53 (*n* = 8)	0.67
Males:				
Metabolic hormones				
Leptin (ng/mL)		32.58 ± 2.26 (*n* = 8)	28.38 ± 2.15 (*n* = 9)	0.20
Insulin (ng/mL)		0.93 ± 0.26 (*n* = 8)	1.30 ± 0.37 (*n* = 9)	0.44
Pituitary hormones				
ACTH (pg/mL)	17.94 ± 3.46 (*n* = 8)		22.05 ± 5.53 (*n* = 9)	0.55
FSH (ng/mL)		3.19 ± 0.67 (*n* = 8)	2.77 ± 0.41 (*n* = 9)	0.59
GH (ng/mL)		0.54 ± 0.09 (*n* = 6)	2.14 ± 0.54 (*n* = 9)	**0.03**
LH (pg/mL)		392.17 ± 166.81 (*n* = 8)	653.28 ± 200.41 (*n* = 9)	0.34
Prolactin (ng/mL)		1.15 ± 0.33 (*n* = 8)	2.07 ± 0.85 (*n* = 9)	0.35
TSH (pg/mL)		173.10 ± 43.22 (*n* = 8)	258.30 ± 53.23 (*n* = 9)	0.24

Boldface indicates significance and italicized text indicates a statistically non-significant trend. Comparisons were made between genotypes of the same sex using unpaired *t*-tests; n = 6–9 per group. All values shown are mean ± S.E.M. Abbreviations: ACTH, adrenocorticotropic hormone; FH, follicle-stimulating hormone; GH, growth hormone; LH, luteinizing hormone; TSH, thyroid-stimulating hormone.

## Data Availability

The data presented in this study are contained within the main article and Supplementary Materials. Inquiries about data should be directed to the corresponding author.

## Supplementary Materials

The following are available online at https://www.mdpi.com/article/10.3390/ijms222011010/s1.

## Abbreviations

Agrp	agouti-related peptide
Arc	arcuate nucleus
BAT	brown adipose tissue
CRH	corticotropin-releasing hormone
DAB	3,3′-diaminobenzidine
ER	endoplasmic reticulum
ERα	estrogen receptor α
FSH	follicle-stimulating hormone
GABA	γ-Aminobutyric acid
GH	growth hormone
DIO	diet-induced obesity
DMH	dorsomedial hypothalamus
HFD	high-fat diet
ME	median eminence
Npy	neuropeptide y
Pomc	pro-opiomelanocortin
RIP	rat-insulin-promoter
ROS	reactive oxygen species
Scly	selenocysteine lyase
SELENOM	selenoprotein M
STAT3	signal transducer and activator of transcription 3
TRH	thyrotropin-releasing hormone
Trsp	selenocysteine tRNA^[Ser]Sec^ gene
UCP1	uncoupling protein-1
WAT	white adipose tissue
