# Supplementary material for: Female Mice with Selenocysteine tRNA Deletion in Agrp Neurons Maintain Leptin Sensitivity and Resist Weight Gain While on a High-Fat Diet
Source: Int J Mol Sci. 2021 Oct 12;22(20):11010. doi: 10.3390/ijms222011010 (PMC8539086; doi:10.3390/ijms222011010)
Supplement: Supplementary file 1 [file ijms-22-11010-s001.zip › ijms-1403629-supplementary.pdf]

International Journal of
Molecular Sciences

MDPI

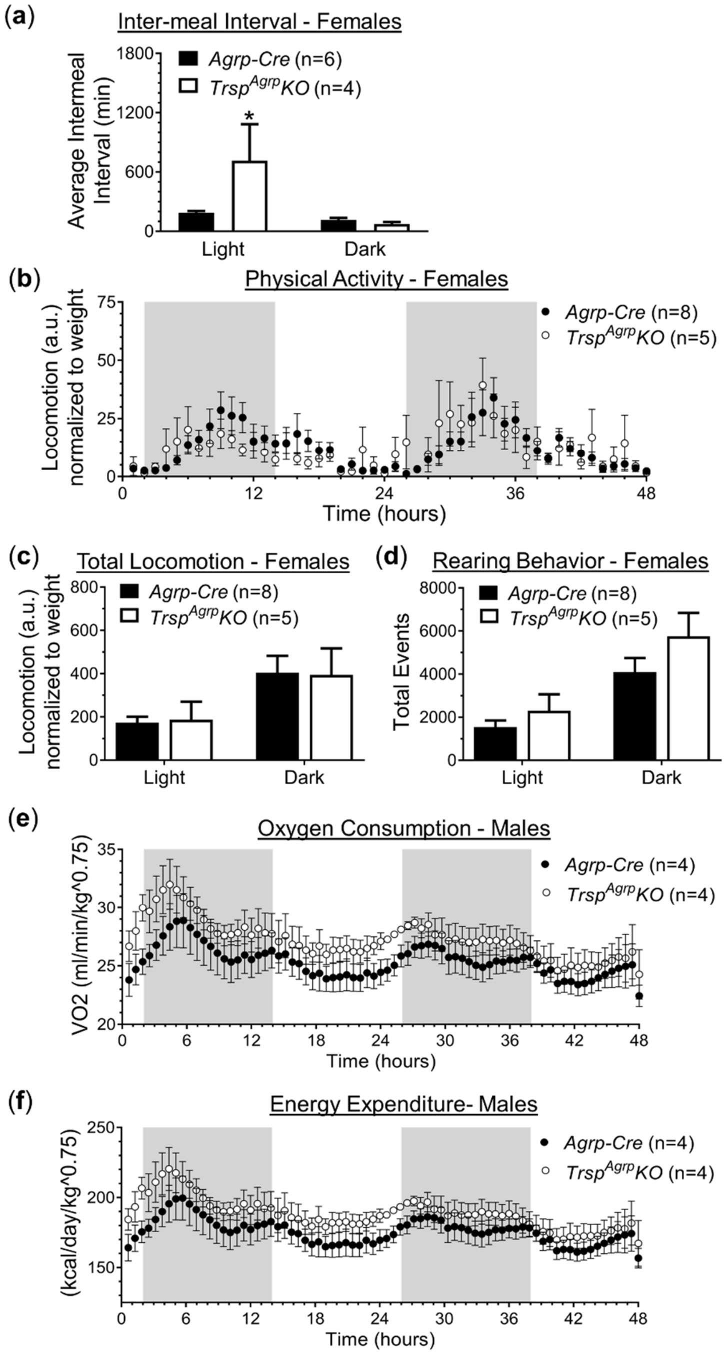

Figure S1. Supplementary metabolic cage data for female and male *Trsp^{Agrp}KO* mice raised on a high fat diet. Meal analysis of food intake data revealed a significantly higher inter-meal interval in female *Trsp^{Agrp}KO* mice compared to *Agrp-Cre* control mice during the light cycle (**a**) (Two-way repeated measures analysis of variance, followed by Sidak's multiple comparisons test: **p*=0.04). Time-course display of physical activity of female *Trsp^{Agrp}KO* mice while in the metabolic cages (**b**). No significant differences between genotypes were detected in terms of either spontaneous locomotor activity (**c**) or rearing events between females of both genotypes (**d**) No significant changes in oxygen

International Journal of
Molecular Sciences

consumption (**e**) or energy expenditure (**f**) were observed in male *Trsp^AgrpKO* mice, although there were slight trends towards an elevation in both parameters. Samples sizes are displayed in the graphs. All values shown are mean ± standard error of the mean.

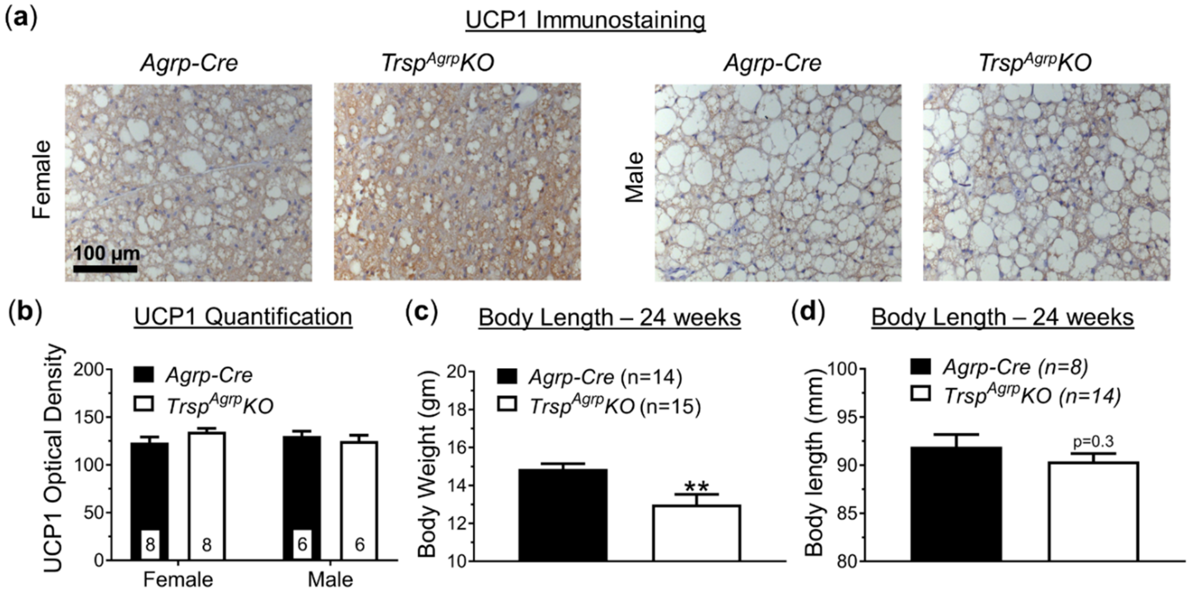

Figure S2. Uncoupling protein-1 **(**UCP1) staining in brown adipose tissue (BAT) sections from *Trsp^AgrpKO* mice of both sexes raised on a high fat diet and supplemental information about female mouse body composition. Sample images of BAT sections stained for UCP1 immunoreactivity taken at 20× magnification. **(a,b)** Optical density measurements did not reveal any significant change due to either genotype or sex. **(c)** Female *Trsp^AgrpKO* mice weighed significantly less than controls at 4 weeks of age (student's test, ** p =0.005) **(d)** and body lengths were comparable at 24 weeks of age (student's test, p = 0.3). Samples sizes are displayed in the graphs. All values shown are mean ± standard error of the mean.
